# Deciphering the mode of action of the processive polysaccharide modifying enzyme dermatan sulfate epimerase 1 by hydrogen–deuterium exchange mass spectrometry†
†Electronic supplementary information (ESI) available: ESI Fig. 1–4 and ESI Table 1. See DOI: 10.1039/c5sc03798k


**DOI:** 10.1039/c5sc03798k

**Published:** 2015-11-30

**Authors:** Emil Tykesson, Yang Mao, Marco Maccarana, Yi Pu, Jinshan Gao, Cheng Lin, Joseph Zaia, Gunilla Westergren-Thorsson, Ulf Ellervik, Lars Malmström, Anders Malmström

**Affiliations:** a Department of Experimental Medical Science , Lund University , Sweden; b Department of Biochemistry , Boston University , Massachusetts , USA . Email: yangmao@sund.ku.dk; c Department of Chemistry , Boston University , Massachusetts , USA; d Department of Chemistry and Biochemistry , Center for Quantitative Obesity Research , Montclair State University , New Jersey , USA; e Department of Chemistry , Lund University , Sweden; f S3IT , University of Zurich , Switzerland

## Abstract

Distinct from template-directed biosynthesis of nucleic acids and proteins, the enzymatic synthesis of heterogeneous polysaccharides is a complex process that is difficult to study using common analytical tools. Therefore, the mode of action and processivity of those enzymes are largely unknown. Dermatan sulfate epimerase 1 (DS-epi1) is the predominant enzyme during the formation of iduronic acid residues in the glycosaminoglycan dermatan sulfate. Using recombinant DS-epi1 as a model enzyme, we describe a tandem mass spectrometry-based method to study the mode of action of polysaccharide processing enzymes. The enzyme action on the substrate was monitored by hydrogen–deuterium exchange mass spectrometry and the sequence information was then fed into mathematical models with two different assumptions of the mode of action for the enzyme: processive reducing end to non-reducing end, and processive non-reducing end to reducing end. Model data was scored by correlation to experimental data and it was found that DS-epi1 attacks its substrate on a random position, followed by a processive mode of modification towards the non-reducing end and that the substrate affinity of the enzyme is negatively affected by each additional epimerization event. It could also be shown that the smallest active substrate was the reducing end uronic acid in a tetrasaccharide and that octasaccharides and longer oligosaccharides were optimal substrates. The method of using tandem mass spectrometry to generate sequence information of the complex enzymatic products in combination with *in silico* modeling can be potentially applied to study the mode of action of other enzymes involved in polysaccharide biosynthesis.

## Abstract 2

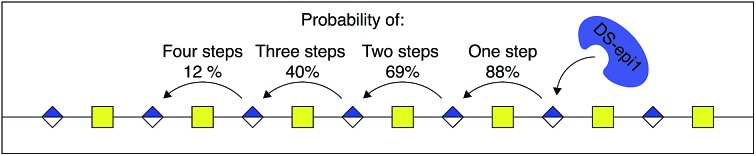
DS-epi1 is a processive enzyme that sequentially epimerizes polysaccharide substrate towards the non-reducing end.

## Introduction

Polysaccharides, such as starch, cellulose, and glycosaminoglycans (GAGs), are basic building blocks of living organisms, together with lipids, nucleic acids, and proteins. Compared with the template-directed biosynthesis of nucleic acids and proteins, the biosynthesis of polysaccharides often involves chain elongation and simultaneous modifications on nascent chains, which are tightly regulated by biosynthetic enzymes and substrate availability. These reactions are often incomplete, resulting in highly heterogeneous products, preventing detailed analysis of the mechanistic behavior of the polysaccharide modifying enzymes. In comparison, the template-directed biosynthesis of nucleic acids and proteins involves processive enzymes that perform many rounds of catalysis before detaching themselves from their substrates.^1^ Largely due to the heterogeneity of the polysaccharide substrates and enzyme products, the mode of action and processivity of the polysaccharide biosynthetic enzymes have rarely been explored.

GAGs are complex linear polysaccharides, common in multicellular organisms, consisting of repeating disaccharide blocks of either hexose or hexuronic acid, linked to a hexosamine. With the exception of hyaluronan, GAGs do not mainly exist as free entities but are parts of proteoglycans (PG), glycoproteins consisting of a core protein with one or several covalently bound GAGs.^2^ Dermatan sulfate (DS) is one of the major GAGs in eukaryotes and its complex template-independent biosynthesis involves at least 21 different enzymes.^3^ After polymerization of the polysaccharide backbone chain, consisting of alternating residues of glucuronic acid (GlcA) and *N*-acetylgalactosamine (GalNAc), the chain modification process ensues. The main chain-modifying enzymes involved in the biosynthesis of DS are dermatan sulfate epimerase 1 and 2 (DS-epi1/2) and dermatan-4-*O*-sulfotranserase 1 (D4ST1). DS-epi1 and DS-epi2 are responsible for catalyzing the inversion of stereochemistry of carbon 5 on GlcA, forming iduronic acid (IdoA), while D4ST1 transfers a sulfate group to the hydroxyl group on position 4 of the GalNAc adjacent to an IdoA, which inhibits back epimerization of the same uronic acid.^4^–^6^ Depending on tissue and core protein, IdoA can be distributed as single units or in blocks. DS-epi2 only forms short IdoA containing blocks, while DS-epi1 together with D4ST1 can produce long blocks of up to 99% of IdoA containing disaccharide units.^5^ Traditionally, a GAG chain containing one or more units of IdoA is called DS, while a chain lacking IdoA is called chondroitin sulfate (CS). However, since very often no pure chain exists, the name CS/DS better describes the hybrid nature of the polymer.

Because DS-epi1, *in vivo*, has been shown to make long stretches of IdoA containing disaccharide units in the CS/DS chains, it is tempting to hypothesize that both DS epimerases work as processive enzymes with different processivity.^6^ However, while the activity and specificity of the DS epimerases have previously been measured *in vitro*, their mode of action and processivity are still not clear.^5^,^7^ Short stretches of IdoA with various sulfation patterns have important functions in binding and controlling the activity of cytokines, growth factors and factors controlling coagulation, including heparin growth factor (HGF), fibroblast growth factor 2 (FGF-2), CXCL13 and heparin cofactor II.^8^ The formation of long stretches of IdoA containing disaccharides have important roles for the regulation of the function of the extracellular matrix, by influencing the fibrillization of collagen.^9^,^10^ This is seen clearly in patients with a loss-of-function mutation in DS-epi1, who have serious malformations of connective tissue in form of the musculocontractural Ehlers-Danlos syndrome.^11^ It is therefore important to develop methods to probe the hypothesized processivity of the DS epimerases.

In this paper, we use DS-epi1 as a model and tested a method combining hydrogen–deuterium exchange LC-MS/MS and *in silico* enzyme–substrate/product interaction simulations to determine the mode of action of polysaccharide enzymes *in vitro*. We found that DS-epi1 attacks a random position of the polymer chain and then sequentially advances towards the non-reducing end and that each additional epimerized disaccharide negatively impacts the affinity between the enzyme and its substrate.

## Experimental

### Cloning and expression of DS-epi1

The part of the human DSE open reading frame (sequence harmonized, Genscript) corresponding to amino acids 23 to 690, 705, 733, 755, 775, 797, 830 and 894 was subcloned together with a C-terminal 8xHIS tag into the NheI and NotI sites of a pCEP-Pu/BM40 (ref. 12) (modified version of pCEP4 from Invitrogen) expression vector using the following primers (Sigma, 5′–3′):

**Table:** 

**Forward** (NheI restriction site in bold letters)	
23	GCATCT**GCTAGC**CTATATTACCGACGAGAACCCAGAGGTCA

**Reverse** (NotI restriction site in bold letters)	
690	GCATCT**GCGGCCGC**GTCAATGGTGATGGTGATGATGGTGGTGGGATGTGGCGATAAACACGTC
705	GCATCT**GCGGCCGC**GTCAATGGTGATGGTGATGATGGTGGTGTCCAGTAGCCTCCCCTGTCCA
733	GCATCT**GCGGCCGC**GTCAATGGTGATGGTGATGATGGTGGTGGGGCACGATAGAGGACTTAATGGC
755	GCATCT**GCGGCCGC**GTCAATGGTGATGGTGATGATGGTGGTGCAGCTGAAAGACAGGCTTGAAATGC
775	GCATCT**GCGGCCGC**GTCAATGGTGATGGTGATGATGGTGGTGCAGCTGAAAGACAGGCTTGAAATGC
797	GCATCT**GCGGCCGC**GTCAATGGTGATGGTGATGATGGTGGTGACTGATGGCGAAAATTCTGTCGATTGC
830	GCATCT**GCGGCCGC**GTCAATGGTGATGGTGATGATGGTGGTGTTCGATCTGTGCAAAAATGTCGG
894	GCATCT**GCGGCCGC**GTCAATGGTGATGGTGATGATGGTGGTGGGGAGCCCTGCTGTGGGTA

The plasmid product was amplified (Library Efficiency DH5α bacteria, Invitrogen), purified (HiSpeed Plasmid Midi Kit, Qiagen) and sequenced (Eurofins MWG Operon).

HEK293 cells (ATCC, CRL 10852) grown in DMEM/F12, GlutaMAX (Life Technologies, catalog number 31331-028) supplemented with 10% FBS (Sigma), 1X PenStrep (Sigma) and 250 μg mL^–1^ G418 (Sigma) were transfected with the plasmid according to protocol from manufacturer (Turbofect, Thermo Scientific). After 48 h the medium was exchanged for selection medium (as above, without G418) containing 2 μg mL^–1^ puromycin (Sigma). Clones expressing the transgene were selected and expanded for approximately two weeks, after which the confluent cell layer was washed with DPBS (Sigma) and the medium exchanged to serum-free DMEM/F12, GlutaMAX containing 0.5 μg mL^–1^ puromycin. Conditioned medium, harvested during a period of several weeks, was clarified by centrifugation and frozen in batches. Clarified medium was applied to an equilibrated HisTrap FF column (GE Healthcare) at a flow rate of 0.5 mL min^–1^, using an ÄKTA Start system (GE Healthcare). The column was washed at 1 mL min^–1^ with a 20 mM pH 7.4 phosphate buffer containing 0.5 M NaCl and 30 mM imidazole, after which the protein was eluted by a 30–300 mM imidazole gradient. Protein purity for each eluted fraction was analyzed by SDS-PAGE on a bis–Tris 4–12% gel in MOPS buffer (Invitrogen), visualized by Brilliant Blue G colloidal (Sigma). Fractions containing pure protein were pooled, buffer exchanged into 20 mM TBS, pH 7.9, on PD-10 columns (GE Healthcare) and concentrated in a 30 kDa MWCO Amicon Ultra centrifugal concentrator (Millipore).

### Preparation and purification of chondroitin oligosaccharides

Defructosylated K4 (dK4, chondroitin) polysaccharide was prepared as described previously.^13^,^14^ Chondroitin (5 mg) was depolymerized by bovine testes hyaluronidase type IV-S (500 U, Sigma-Aldrich) in a Na-acetate buffer (500 μL, 100 mM, pH 5.0) supplemented with NaCl (150 mM), for 1 h at 37 °C. The oligosaccharide products were purified on a Superdex Peptide column (GE Healthcare), using NH_4_HCO_3_ (0.2 M) as eluent, followed by a second round of purification on a TSKgel Amide-80 column (Tosoh Bioscience LLC), using an ammonium formate/acetonitrile gradient as eluent. The purified oligosaccharides were dried in a vacuum concentrator and stored at room temperature until use.

### Determination of DS-epi1 activity

Epimerase activity was measured as previously described, with slight modifications.^4^ Briefly, DS-epi1 (1 μg) was incubated with 30 000 dpm [5-^3^H]dK4 (∼20 μM HexA) in 100 μL 20 mM MES, pH 5.5, buffer supplemented with 2 mM MnCl_2_. After 1 h the sample was boiled and subsequently centrifuged at 20 000*g* for 5 min. The supernatant was distilled and tritium release was quantified with liquid scintillation counting.

### Incubation of oligosaccharides with DS-epi1

The assay buffer for DS-epi1, MES (20 mM, pH 5.5) supplemented with MnCl_2_ (2 mM), was lyophilized and reconstituted in D_2_O (99.9 atom% D, Sigma-Aldrich). Dried oligosaccharides (5 μg) were dissolved in the D_2_O assay buffer and incubated for 20 h at 37 °C together with DS-epi1 (0.2 μg) and BSA (100 μg). The final concentration of D_2_O was ∼97% due to addition of enzyme and BSA (3 μL to a final volume of 100 μL). After quenching the incubation by boiling, the samples were centrifuged and the supernatant was purified on an Amide-80 column, as above. Fractions containing the oligo of interest were pooled and dried in a vacuum concentrator.

### Mass spectrometric analysis of oligosaccharide products

Purified oligosaccharide products were labeled with *O*-(pyridin-3-ylmethyl)hydroxylamine, a sequestered proton reagent for acid-catalyzed glycan sequencing (PRAGS), as previously described.^15^ Briefly, about 1 nmol of dried oligosaccharide products were dissolved in 12 μL 1% acetic acid and mixed with 3 μL of a 30 mM solution of PRAGS reagent in acetonitrile. After incubation at 37 °C for overnight (∼16 h), the labeled oligosaccharides were separated from the remaining excess reagent and salts on a Superdex Peptide PC 3.2/30 column (GE Biosciences, Piscataway, NJ) equilibrated with 25 mM ammonium acetate, 5% acetonitrile.

The desalted oligosaccharides were dissolved in 5% isopropanol, 0.1% ammonia to a final concentration of 10 pmol μL^–1^ and directly infused into a 12 T solariX™ hybrid Fourier Transform Ion Cyclotron Resonance (FT-ICR) mass spectrometer (Bruker Daltonics, Bremen, Germany) using an Apollo II nanoESI source. The instrument was operated in the negative mode and precursor ions of interest were isolated with an isolation window of 7 Th. Collision induced dissociation (CID) was carried out in the hexapole collision cell.

### Simulations of enzyme–substrate/product interactions

*In silico* simulations of two simple interaction principles (IP) between the enzyme and its substrate were carried out to facilitate the interpretation of the mass spectrometry data. The two interaction principles were: (i) IP-I random attack and sequential progression toward the non-reducing end and (ii) IP-II attack on the non-reducing end and sequential progression toward the reducing end. For each interaction principle, we optimized one or two parameters using a brute-force parameter sweep approach, scoring with a simple correlative function described in the next section. Due to the relative small amounts of data, we reduced the number of parameters by assuming the following:

(1) Each oligomer could only interact a single time with a single enzyme.

(2) Each interaction was allowed to proceed to completion and always concluded with the enzyme and its substrate detaching.

(3) The first dimer from the reducing end was excluded from the potential attack in agreement with the experimental data.

(4) The enzyme could only move in a single direction.

(5) Only the ratio between progression and dissociation was considered.

#### IP-I

Random attack and progression towards non-reducing end. Two parameters were fitted – the ratio between moving along the chain or detaching (off_rate) and how that ratio changed with increased number of epimerized dimers (delta-off_rate). Off-rates (20–0) and delta-off-rates (100–70) were parameter swept in a brute-force manner. 1000 oligomers were created for each parameters pair for a total of 651 000 *in silico* oligomers.

#### IP-II

Attack on the non-reducing end and progression towards the reducing end. Single parameters fitted – the ratio between moving along the chain and dis-attaching (off_rate). 1000 oligomers were created for each value for off-rate (60–1) for a total of 60 000 *in silico* oligomers.

### Score model to assess similarity between a set of *in silico* oligomers with the experimental MS data

The *in silico* oligomers were used to compute theoretical values for the distribution of the number of epimerized dimers per oligomer for the various oligomer lengths and the rate of epimerization for the several fragments observed in the mass spectrometer. The correlation between the theoretical distribution and the observed distribution was calculated for eight separate distributions and the optimal model can hence achieve a score of 8. The scores considered were seq_score, oligomer12_score, oligomer14_score, oligomer16_score, oligomer18_score, frag9_score, frag11_score and frag13_score; seq_score is based on the sequencing results from the dp14 chondroitin oligosaccharide and corresponds to the number of epimerizations as a function of the dimer position from the reducing end, the four oligomer scores corresponds to the distribution of epimerization events for each length of oligomer (dp12–18) and the frag scores corresponds to the distribution of epimerization events for the Y fragments of dp14.

## Results and discussion

### Expression of active recombinant DS-epi1

**Fig. 1:**
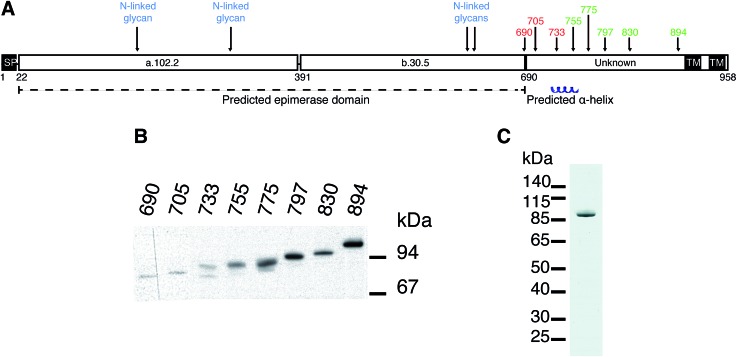
DS-epi1 with a minimum length of 755 is required for activity. (A) Predicted structure of DS-epi1. Numbers above the schematic representation show enzymatically active (green) and inactive (red) recombinant expression products, all starting from amino acid 23. SP = signal peptide. TM = transmembrane region. a.102.2 = predicted α-helix rich domain. b.30.5 = predicted β-sheet rich domain. (B) Western blot of expression products. (C) Coomassie Brilliant Blue G stained SDS-PAGE gel of purified DS-epi1-775.

Several different plasmid constructs, ending between amino acid 690 and 894, were prepared for DS-epi1 in order to examine the length necessary for an active enzyme product (Fig. 1). Constructs ranging from amino acid 690 to 733 were inactive, while those from amino acid 755 were active. Remarkably, it was found that construct 23–775 gave a protein yield approximately 50 times higher than the other constructs. This construct was therefore used in downstream experiments and after affinity purification the protein yield of DS-epi1 775 was approximately 4 mg l^–1^ growth medium.

The HEK293-EBNA episomal expression system proved to yield excellent amounts of secreted recombinant, eukaryotic posttranslationally modified, protein. Earlier expression attempts in *E. coli* and *S. cerevisiae* (data not shown) gave rise to an inactive enzyme. As we have previously shown, all predicted *N*-glycans in DS-epi1 are present when the protein is expressed in HEK 293 cells, and necessary for optimal enzymatic activity.^16^ Also, attempts to express DS-epi2, C4ST1 and D4ST1 in bacteria yielded either no expression or the expression of inactive products in inclusion bodies (results not shown). In contrast, almost all heparan sulfate biosynthetic enzymes have been expressed successfully in prokaryotic expression systems, suggesting different roles of the *N*-glycans in the two different biosynthetic machineries.^17^

At the border between the longest inactive (amino acid 23–733) and the shortest active (amino acid 23–755) product a predicted α-helix (amino acid 736–764) is situated. While we have previously suggested the structure and catalytic amino acids of the epimerase domain (amino acid 23–690) of DS-epi1, the structure and role of the C-terminal domain (amino acid 691–958) remains unknown. Several explanations for the necessity of the C-terminal domain are possible, including formation of multimeric complexes, correct protein folding, and/or locking the substrate in place during catalysis. No homology can be found between the C-terminal domain and other proteins, but crystallization and subsequent structure solving could give valuable information regarding its role. One construct, DS-epi1 775, gave much higher expression efficiency than the others. This behavior can be due to several reasons, including protein solubility and stability as well as mRNA structure and stability.^18^

### Hydrogen–deuterium exchange mass spectrometry enables analysis of heterogeneous epimerase products

**Fig. 2:**
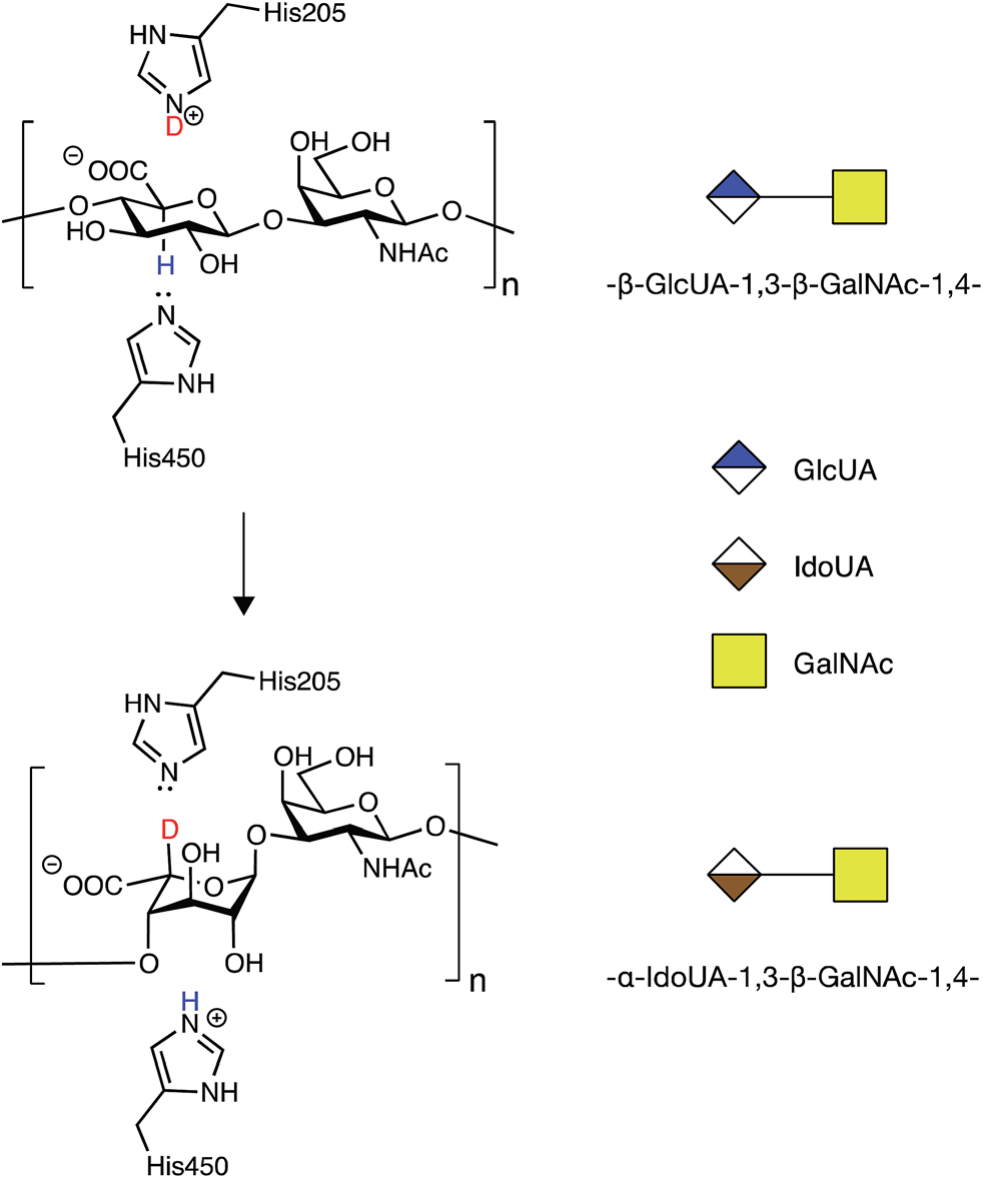
Epimerization of GlcA to IdoA by DS-epi1 conducted in D_2_O (modified from^16^). First, a hydrogen on C5 of GlcA is removed by His450 of DS-epi1. Next, a deuterium is incorporated from His205 of DS-epi1 into an intermediate product (not shown), generating IdoA.

In order to address the mode of action and processivity of DS-epi1, we first envisioned a strategy that would allow us to feed the enzyme with purified oligosaccharide substrates and analyze the pattern of DS-epi1-modified sites after reaction. Traditionally, the *in vitro* activity of DS-epi1 is gauged by the release of tritium from tritium-labeled chondroitin substrates.^14^ However, the total radioactivity release cannot be used to quantitatively measure the attacked sites, given the reversibility of the reaction. Taking advantage of the hypothesized elimination/addition mechanism for this type of epimerases (Fig. 2), a previous study used MS to successfully monitor the incorporation of heavy atoms to substrates by heparan sulfate C5 epimerase, when the reaction was carried out in D_2_O.^19^,^20^

**Fig. 3:**
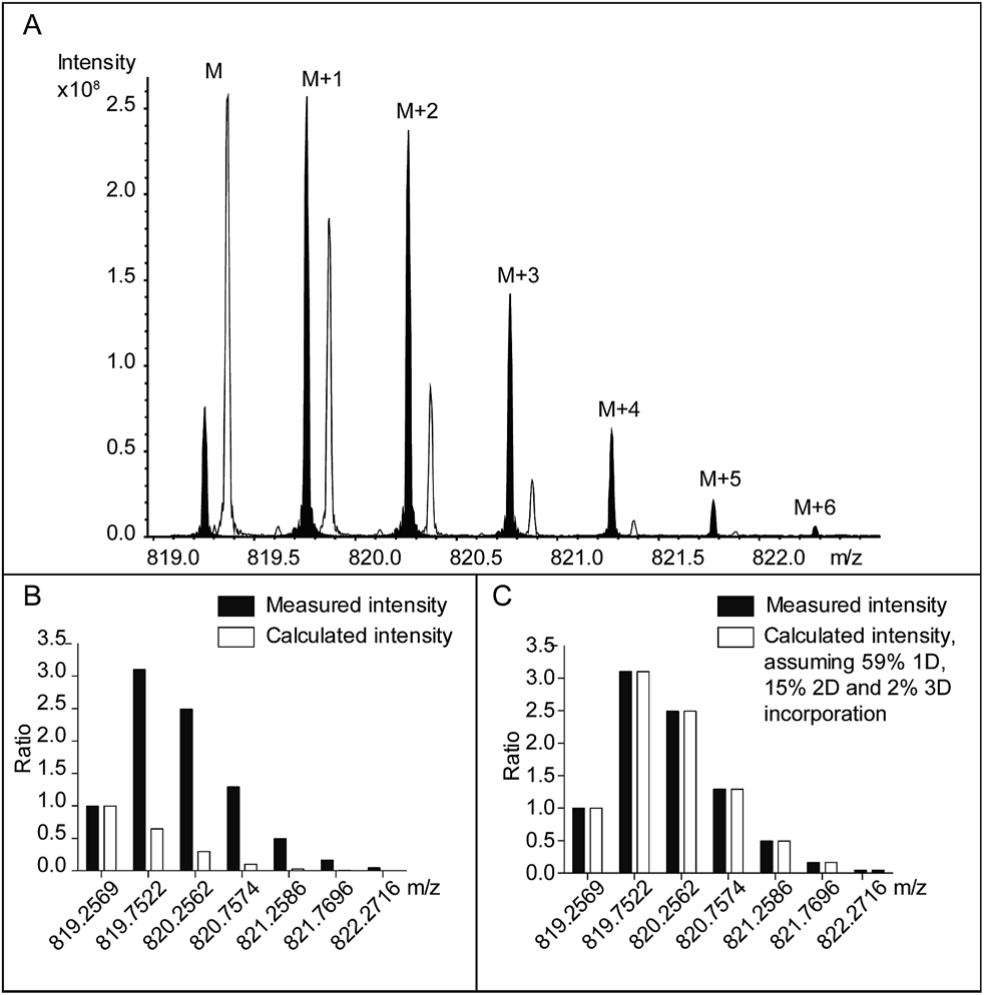
(A) The [M – 2H]^2–^ isotopic clusters of a PRAGS (see “Experimental”) labeled chondroitin dp8 oligosaccharide following incubation with DS-epi1 in a H_2_O-containing buffer (no fill), and in a D_2_O-containing buffer (black fill). For clarity, the peaks from the H_2_O incubation have been mass shifted manually. The data were normalized and compared to the theoretical isotopic distribution pattern, assuming either (B) no deuterium incorporation or (C) corrected with 59% one deuterium, 15% two deuterium and 2% three deuterium incorporation, which resulted in the best fit to the experimental data.

In combination with HPLC separation of IdoA- and GlcA-containing disaccharides, the previous study correctly measured the epimerase activity, *i.e.* the number of catalytic cycles. The first aim of our study was to extend the capability of the method to measure both the number and the position of the DS-epi1 modified sites in a complex product mixture. Compared to the radioactivity release method, the hydrogen–deuterium exchange experiment leaves traceable stable isotopic labels on the modification sites, which can be analyzed by high-resolution mass spectrometry. Subsequently, by comparing the abundances of isotopic species in the modified oligosaccharides with their natural abundances, the numbers of modified uronic acid sites can be calculated. To test our hypothesis, oligosaccharide substrates ranging from degree of polymerization (dp) 4 to dp10 were purified and incubated with DS-epi1 in D_2_O. As expected, DS-epi1-catalyzed incorporation of deuterium into the oligosaccharide substrates caused a discernible shift of the isotopic pattern in the mass spectra (Fig. 3A shows the incorporation data for a dp8).

The relative abundances of the M + 1 isotopic species were greatly enhanced in spectra obtained from oligosaccharides incubated in a D_2_O-containing buffer compared with oligosaccharides incubated in a H_2_O-containing buffer (Fig. 3B). The abundances of the oligosaccharide substrates that incorporated one deuterium (1D) were calculated by subtracting the calculated M + 1 intensities resulting from the natural occurrence of the M + 1 isotopic species from the measured values.

Similarly, the calculated M + 2 peak intensities of the unmodified oligosaccharides and the calculated M′ + 1 peak intensities of the 1D-incorporated oligosaccharides were subtracted from the measured M + 2 values to get the abundances of the oligosaccharide substrates that incorporated two deuterium (2D). The calculation continued until no extra isotopic species needed to be explained and the measured isotopic patterns matched the calculated ones (Fig. 3C). Depending on the length of the oligosaccharide, between one and four deuterium atoms were incorporated (Table 1).

**Table 1:** Frequency and number of modifications in the complex mixture of oligosaccharide products. Percent of oligosaccharide products incorporating one, two, three or four deuterium

	1 D	2 D	3 D	4 D	Number of HexA	Degree of modification/HexA
dp4	22%				2	11%
dp6	36%	7%			3	17%
dp8	59%	15%	2%		4	24%
dp10	42%	31%	9%	2%	5	28%

**Fig. 4:**
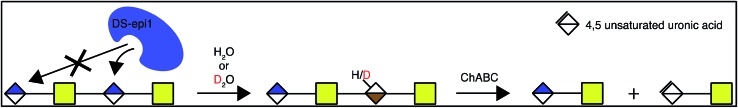
The non-reducing end uronic acid is not attacked by DS-epi1. Chondroitin oligosaccharide dp4 was incubated with DS-epi1 in either a D_2_O or an H_2_O containing buffer. The saccharide products were then depolymerized with chondroitinase ABC (ChABC), separated by HPLC and analyzed by MS to determine the mass distribution of the products.

We observed DS-epi1-catalyzed incorporation of deuterium atoms into oligosaccharide substrates as short as dp4. To determine if there was a preference in position of the attacked uronic acid, tetrasaccharides incubated with DS-epi1 were digested with chondroitinase ABC, followed by HPLC separation of the saturated and non-saturated disaccharide products and MS analysis. Both disaccharide products displayed normal isotope distribution patterns, showing that neither the unsaturated nor the saturated disaccharide contained deuterium (Fig. 4 and ESI S1†). This proved that the internal uronosyl residue was the only uronic acid that had been attacked by the enzyme during the reaction, as the deuterium was abstracted by chondroitinase ABC during the digestion.

### Optimal substrate binding requires an octasaccharide or longer

Next, we determined the substrate length that the enzyme bound preferentially by considering the percentage of oligosaccharides in each length group (*i.e.* dp4, dp6 *etc.*) that incorporated one deuterium atom and the overall incorporation of deuterium per uronic acid residue (Table 1). For smaller substrates one uronic acid in the chain was clearly favored by the enzyme, resulting in the most abundant products with one deuterium incorporated. For longer oligosaccharides, the enzyme bound equally well to two or more uronic acids in the chain, the percentage of oligosaccharides with only one deuterium decreased, and the overall degree of modification per uronic acid reached a plateau. The results showed that the minimal size for optimal substrate binding was an octasaccharide. This observation was in agreement with the results from the traditional tritium-releasing experiments (Fig. ESI S2†), confirming the robustness of the hydrogen–deuterium exchange strategy. GAG octasaccharides have the capacity to interact with growth factors, such as fibroblast growth factor 10 (FGF-10), and viruses, such as herpes simplex.^21^,^22^ Further, sulfotransferases involved both in heparin and dermatan sulfate biosynthesis have previously been shown to accept octasaccharides as substrates in enzyme assays.^23^,^24^

### Generation of site-specific modification information by acid-catalyzed glycan sequencing (PRAGS)

**Fig. 5:**
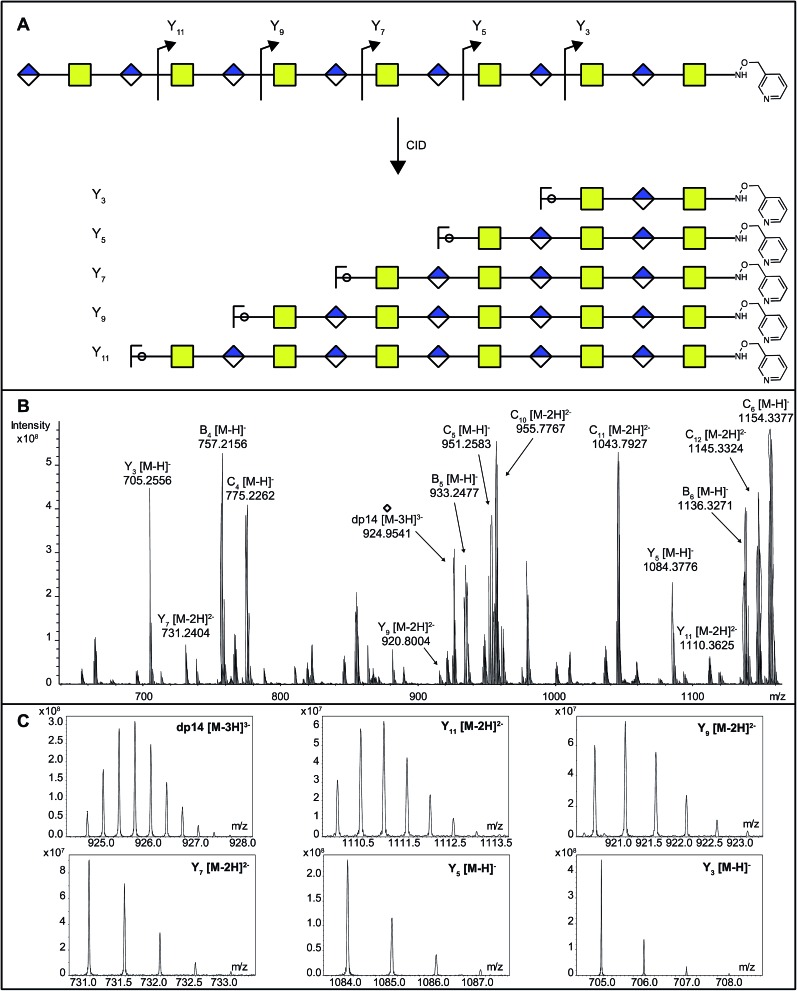
The position and degree of modification of uronosyl residues modified by DS-epi1 can be measured from the CID fragmentation of PRAGS-labeled oligosaccharide products. (A) Scheme of CID fragmentation of a PRAGS-labeled chondroitin dp14 oligosaccharide. (B) The mass spectra of the 20 V CID mass spectra of the dp14 oligosaccharide incubated with DS-epi1 in a D_2_O-containing buffer. (C) The isotopic distribution patterns of the dp14 precursor ion and its fragments were used for deuterium incorporation calculations. Note the skewed isotopic pattern, especially in the full-length oligosaccharide and the longer fragments, due to deuterium incorporation.

As DS-epi1-catalyzed incorporation of deuterium atoms leave the modified uronic acids with stable isotopic marks, we next sought to get site-specific modification information on the oligosaccharide products by tandem mass spectrometry. One potential pitfall with tandem mass spectrometry of polysaccharides is the high degrees of symmetry in these molecules. Since polysaccharides are generally unmarked along their sequences, lacking side chains as found in peptides, C-type fragment ions from the non-reducing ends may have the same elemental compositions as Y-type fragment ions from the reducing ends.^25^ To break this undesired symmetry of the DS-epi1 modified oligosaccharides, we labeled the reducing end aldehydes of oligosaccharides with a derivatization reagent designed for acid-catalyzed sequencing (PRAGS).^15^ We found that the PRAGS labeled saccharides produced systematic and predictable glycosidic bond cleavages by CID, which generated reducing-end Y-type product ions (Fig. 5).

**Fig. 6:**
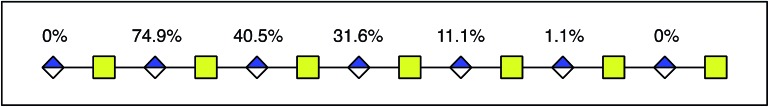
Modified uronic acids accumulated toward the non-reducing end. The figure shows a dp14 product, where the number above each uronic acid represents the degree of modification. For each uronic acid, the degree of modification was calculated by subtracting the total degree of modification of fragment Y_*X*–2_ from the total degree of modification of fragment Y_*X*_.

In order to dissect the mode of action and processivity of DS-epi1, we analyzed the pattern and order of modification sites on the oligosaccharide products. Longer oligosaccharides were analyzed (dp12–dp18, Fig. ESI S3†); a chondroitin dp14 was selected for further analyses because it was the longest oligosaccharide that we could get a complete series of Y-type fragments by CID. As breaking the glycosidic bond to the non-reducing end of the uronic acid residue risks losing the DS-epi1-incorporated deuterium during the fragmentation, we only selected the odd-numbered Y-type fragment ions to avoid potential error to our calculation. Since it was demonstrated above that the non-reducing end uronic acid was not modified, the full-length dp14 precursor in the spectra was used *in lieu* of the low abundant Y_13_ ion. The extent of deuterium incorporation on the selected Y-type fragment ions was similarly calculated as was done for the precursor ions described above and summarized in Table 2. A clear accumulation of modified sites could be seen towards the non-reducing end of the sequenced dp14 product (Fig. 6).

**Table 2:** Content of deuterium in the various fragments of a chondroitin dp14 oligosaccharide after incubation with DS-epi1 in D_2_O

	0 D	1 D	2 D	3 D	4 D
Y_3_	100%				
Y_5_	99%	1%			
Y_7_	88%	12%			
Y_9_	64%	28%	8%		
Y_11_	40%	37%	21%	2%	
dp14	19%	30%	31%	15%	5%

### *In silico* modeling of site-specific information reveals a processive reducing to non-reducing end mode of action of DS-epi1

**Fig. 7:**
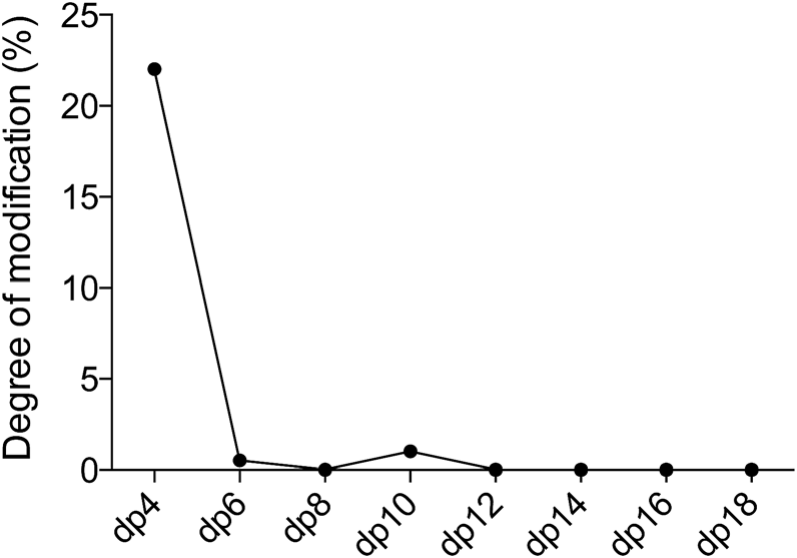
The reducing end uronic acid is a poor substrate for oligosaccharides longer than dp4. The figure shows the percentage of deuterium incorporated into the reducing end uronic acid for various oligosaccharides.

When the sequence of the bulk of modified dp14 products was known it was possible to move forward with simulations in order to reveal how DS-epi1 had modified its substrate. The theoretical product-yield after incubation of a chondroitin dp14 oligosaccharide with DS-epi1 is 128 (2^7^) different epimer products; however as experimental constraints increased, the number of possible products decreased. For example, in the previous section it was determined that the non-reducing end uronic acid was not epimerized and that for a substrate longer than dp4 the amount of deuterium incorporated into the reducing end uronic acid was negligible (Fig. 7). Two simple interaction principles (IP-I and IP-II) were proposed for the mode of action of DS-epi1 on chondroitin. IP-I constituted a *modus operandi* where the enzyme would attack randomly on the oligomer and then act processively from the reducing to the non-reducing end. Next, based on the fact that a clear accumulation of modified sites could be seen towards the non-reducing end of the sequenced dp14 oligosaccharide, IP-II included an attack on the non-reducing end followed by a processive action towards the reducing end.

*In silico* oligomers were generated by simulating enzyme–oligomer interactions, as described in the method section. A total of 711 models were generated, out of which the top 509 highest scoring models belonged to IP-I (ESI Table 1†). The global score for the highest scoring IP-I was 7.34 for the optimal off-rate of 12% and a delta off-rate of 10% for each additional event and the score for the highest scoring IP-II was 6.54 for an optimal off-rate of 47%.

**Fig. 8:**
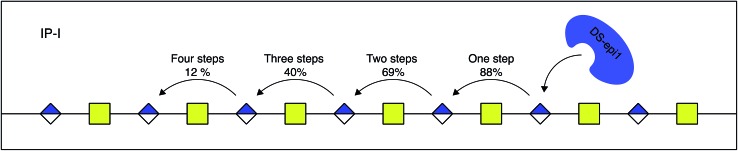
DS-epi1 acts processively from reducing to non-reducing end. The probability of additional modification steps is shown, calculated by the off-rate and the delta off-rate for IP-I.

The relative difference in score between the two highest scoring models for each interaction principle was small, but since IP-I had higher scores both for the oligomer sub scores and the fragment sub scores (ESI Table 1†), we concluded that the mode of action of DS-epi1 is a concerted one, going from the reducing towards the non-reducing terminal (Fig. 8).

**Fig. 9:**
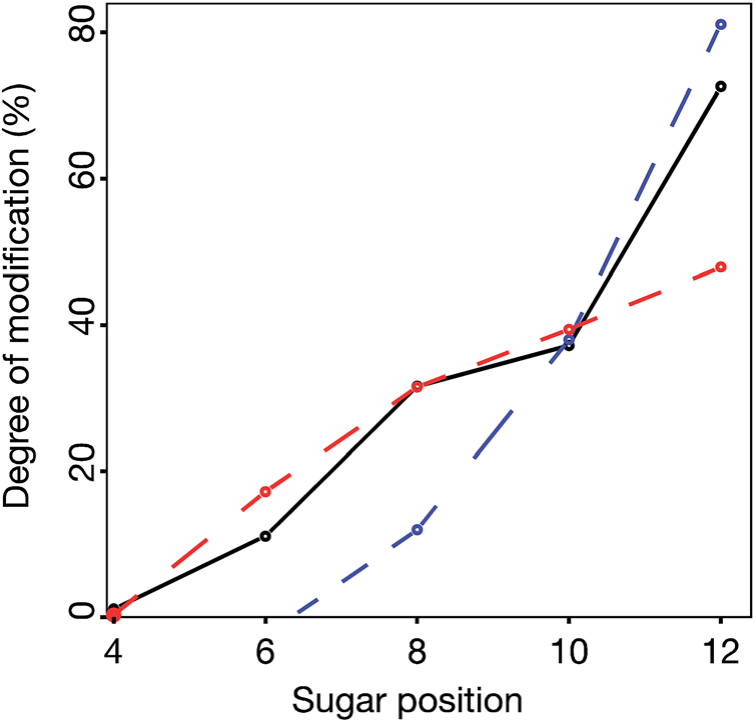
Comparison between experimental (black solid line) and model data for the best scoring models from IP-I (red dashed line) and -II (blue dashed line), showing the accumulated degree of modification of uronic acids (counted from reducing end) in a chondroitin dp14 oligosaccharide.

Statistics from the processive reducing to non-reducing end interaction principle was compared to experimental data in three ways – the total degree of epimerization as a function of distance from the reducing end in the dp14 product (Fig. 9) and the number of epimerized uronic acids per oligomer and per fragment of dp14 (Fig. ESI S4†). For all comparisons the interaction IP-I calculations had a high correlation with experimental data.

Using the results from the simulations, it could be shown that for each epimerization, the enzyme binds less efficiently to the substrate and after approximately four to five events it releases the GAG chain. It is well known that IdoA has a less rigid structure than GlcA.^26^ An explanation for the negative impact on the enzyme–substrate affinity when DS-epi1 modifies its substrate could be that the conformation of IdoA increases the polymer curvature, which in turn could make the interaction surface smaller. As a consequence, short stretches of the GAG chains are isomerized. Further on, the different curvature of the GlcA stretches, compared to the IdoA stretches, may be important for subsequent modifications or for the biological activity of CS/DS in, for example, binding of the fibroblast growth factor family, HGF and heparin cofactor II.^27^–^29^

Our data supports the conclusion that the mode of action of DS-epi1 is from the reducing towards the non-reducing terminal, which is the opposite direction that a previous study proposed for another GAG biosynthetic enzyme: heparan sulfate (HS), *N*-deacetylase/*N*-sulfotransferase 1 (NDST-1).^30^,^31^ Like DS-epi1, NDST-1 is the first modifying enzyme that works on the nascent HS polysaccharide chains. Also, NDST-1 is proposed to play a dominating role in forming highly sulfated N–S domains in HS, which is similar to the role of DS-epi1 in making the IdoA-rich domains in CS/DS. It is therefore significant that different enzymes have evolved to different mode of actions when playing similar roles in modifying nascent polysaccharide chains. Considering that the downstream biosynthetic enzymes are likely relying on actions of either NDST-1 or DS-epi1 and may even physically associate with either of them, our result suggests that the overall biosynthetic mechanism of CS/DS could be very different from that of HS, although both of them are categorized as GAGs.

Polysaccharide processivity opens up the possibility for an efficient formation of long stretches of IdoA, which have been shown to be of major importance for CS/DS regulatory effect on collagen fibrillization.^9^,^10^ However, DS-epi1 itself does not have the capacity to generate IdoA blocks longer than four to five disaccharides units, as shown here. In order for that to occur, both *in vitro* and *in vivo*, 4-*O* sulfation by dermatan 4-sulfotransferase 1 (D4ST1) is needed.^6^,^32^,^33^ Future experiments are therefore necessary to assess the processivity of DS-epi1 in company with D4ST1.

Three families of enzymes epimerize the hexuronyl C5 atom at the polymer level: the heparan sulfate epimerase, the dermatan sulfate epimerases and the alginate epimerases.^34^ Up until now, only the alginate epimerase AlgE4 has been shown to act processively on its substrate, but the described methodology can be applied to all of these enzymes and might shed light on the regulation of their modes of action.^35^ However, it is worth mentioning that the described methodology is not limited to the study of polysaccharide epimerases, which rely on hydrogen–deuterium exchange to generate mass differences in the enzyme products. Many polysaccharide-modifying enzymes, such as sulfotransferases, introduce mass changes to the substrates by their own actions. In those cases, information on the site-modification can be readily obtained by tandem mass spectrometry on a complex product mixture, which may or may not be isolated into single product components. The processivity and mode of action can be mathematically modeled using the complex information generated by tandem mass spectrometry.

## Conclusions

In this paper we have described a tandem MS technique based on fragment analysis of reducing-end labeled GAG oligosaccharides that have been incubated in D_2_O, to obtain information about the enzyme modification sites in a complex mixture of enzyme products. By using this technique, in combination with *in silico* modeling, we have determined that DS-epi1 acts as a processive enzyme that sequentially epimerizes GlcA to IdoA from the reducing towards the non-reducing end. This methodology can be potentially applied to study the mode of action of other enzymes involved in polysaccharide biosynthesis.

## Supplementary Material

Supplementary information

Supplementary information
