# Supplementary material for: Deciphering the mode of action of the processive polysaccharide modifying enzyme dermatan sulfate epimerase 1 by hydrogen–deuterium exchange mass spectrometry
Source: Chem Sci. 2015 Nov 30;7(2):1447–56. doi: 10.1039/c5sc03798k (PMC4755500; doi:10.1039/c5sc03798k)
Supplement: Supplementary file 1 [file SC-007-C5SC03798K-s001.pdf]

Electronic Supplementary Material (ESI) for Chemical Science.
This journal is © The Royal Society of Chemistry 2015

Supporting information

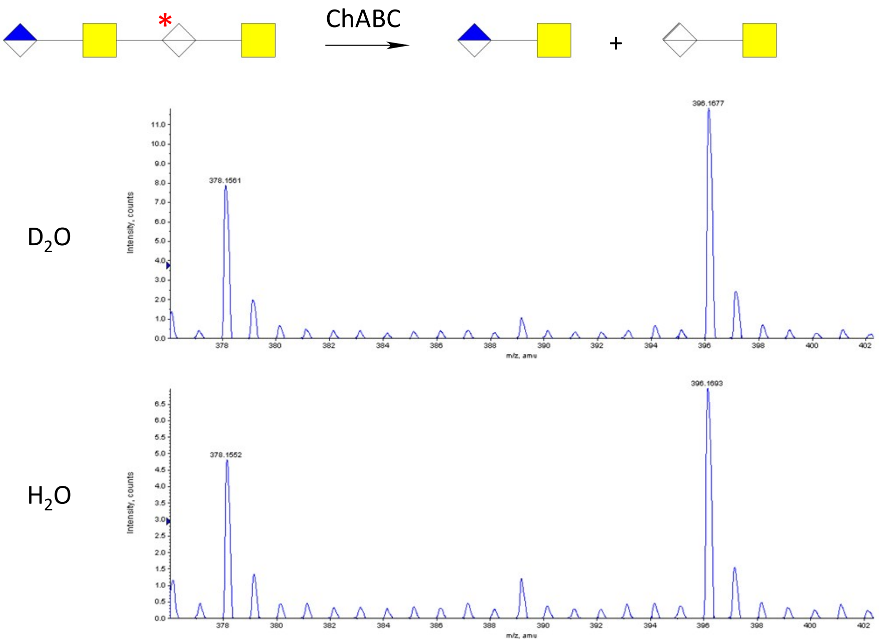

Figure S1. The non-reducing end uronic acid is not modified. Chondroitinase ABC digestion products of a chondroitin tetrasaccharide incubated with DS-epi1 were analyzed by mass spectrometry. Products from samples incubated in both a D_2O buffer and a H_2O buffer displayed normal isotopic distribution patterns, showing that the non-reducing end uronic acid was not modified.

DS-epi1 acts on a tetrasaccharide but requires an octasaccharide for optimal binding

To assay for the DS-epi1 substrate efficiency, incubations were made with equimolar amounts (based on uronic acid) of C5-^{3}H-radiolabelled chondroitin oligosaccharides from dp4 (dp=degree of polymerization) to dp10. When the uronic acid is epimerized, tritium is released into the assay buffer and the activity can be directly correlated to the amount of tritium recovered by distillation of the assay mixture. The results show that the tetrasaccharide functioned as a substrate, even though the activity was low. The release of tritium increased to reach a plateau at dp8, suggesting that an octasaccharide was the shortest oligomer required for optimal binding in the active site.

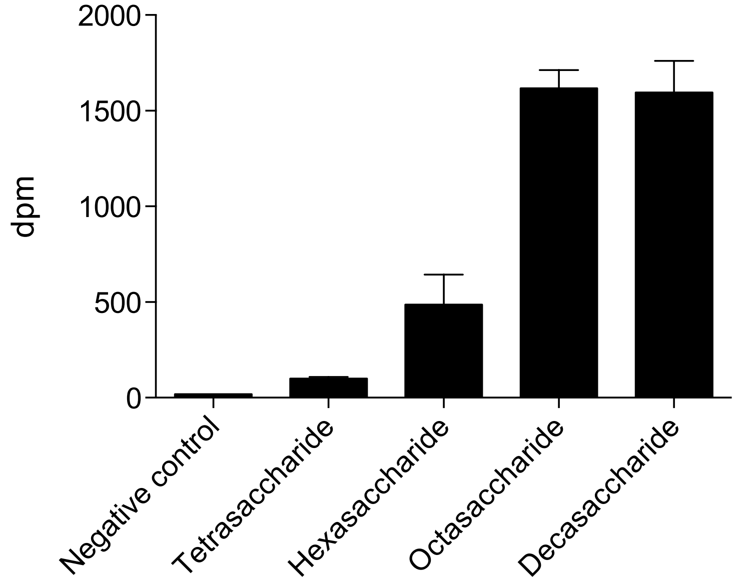

Figure S2. Release of tritium from C5-^{3}H-dK4 oligosaccharides ranging from dp4-dp10.

A

dp12, D_2O, PRAGS labeled
Measured vs. calculated intensity,
Modeled with 38.2 % 1D, 27.0 % 2D, 9.8% 3D, 0.6% 4D incorporation

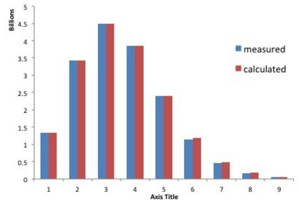

B

dp14, D_2O, PRAGS labeled
Measured vs. calculated intensity,
Modeled with 29.8 % 1D, 29.0 % 2D, 17.8% 3D, 3.1% 4D incorporation

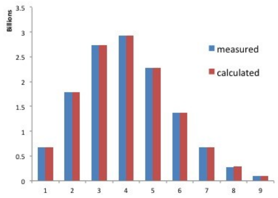

C

dp16, D_2O, PRAGS labeled
Measured vs. calculated intensity,
Modeled with 25.1% 1D, 27.3% 2D, 20.7% 3D, 7.9% 4D , 0.7% 5D incorporation

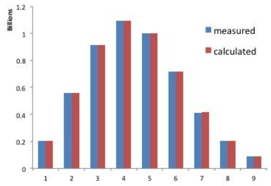

D

dp18, D_2O, PRAGS labeled
Measured vs. calculated intensity,
Modeled with 23.9% 1D, 25.4% 2D, 20.2% 3D, 7.9% 4D , 2.7% 5D incorporation

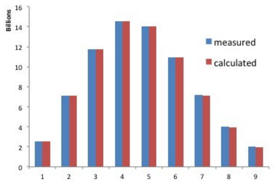

Figure S3. Deuterium incorporation analysis of dp12-dp18 (A-D) incubated with DS-epi1 in D_2O.

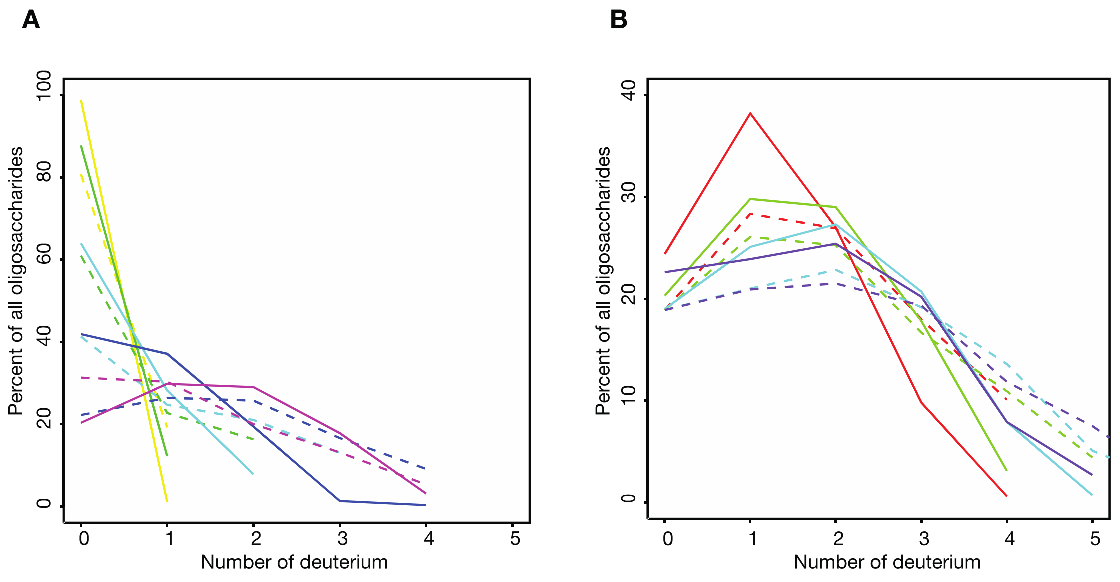

Figure S4. The percentage of 0, 1, 2, 3, 4 and 5 deuterium is shown for **A** fragments of a dp14 oligosaccharide (Y5: yellow, Y7: green, Y9: light blue, Y11: dark blue, dp14: violet) and B oligosaccharides dp12-dp18 (dp12: red, dp14: green, dp16: light blue, dp18: violet). Experimental data is solid line and model data is dashed line.
